# Case Report: Pharmacokinetics of ceftazidime and avibactam during and after CRRT in an elderly patient and their associations with CNS adverse effects

**DOI:** 10.3389/fphar.2025.1569715

**Published:** 2025-05-21

**Authors:** Haiying Huang, Yun Han, Yinshan Wu, Feng Guo, Zhenwei Yu

**Affiliations:** ^1^ Intensive Care Unit, Sir Run Run Shaw Hospital, School of Medicine, Zhejiang University, Hangzhou, China; ^2^ Research Center of Clinical Pharmacy, Sir Run Run Shaw Hospital, School of Medicine, College of Pharmaceutical Science, Zhejiang University, Hangzhou, China

**Keywords:** ceftazidime/avibactam, continuous renal replacement therapy, pharmacokinetic, electroencephalogram, adverse events

## Abstract

Ceftazidime/avibactam is effective for treating infections caused by multidrug-resistant gram-negative bacteria and is widely used. The pharmacokinetic data of ceftazidime and avibactam in patients receiving continuous renal replacement therapy (CRRT) are limited. It is challenging to dose ceftazidime/avibactam, as excessive exposure is associated with central nervous system (CNS) adverse events, especially in older patients. This case reported the pharmacokinetic parameters of ceftazidime and avibactam (1.25 g every 8 h) in an elderly patient during and after CRRT (continuous veno venous hemofiltration mode), which were estimated based on a first-order elimination equation and a two-point sampling strategy. CRRT accounted for 84.9% of the total clearance rate of ceftazidime and 77.1% of the total clearance rate of avibactam. Excessive drug exposure (plasma concentrations of ceftazidime and avibactam were 109 and 20.6 mg/L, respectively) 5 days after discontinuation of CRRT resulted in adverse CNS reactions, which manifested as involuntary convulsions and abnormal brain discharge. This case study provides pharmacokinetic data of ceftazidime and avibactam in patient during and after CRRT and information about the possible relationship between concentrations and CNS adverse reactions.

## 1 Introduction

Ceftazidime/avibactam is a novel β-lactam/β-lactamase inhibitor antibiotic used to address the increasing prevalence of multidrug-resistant (MDR) gram-negative bacteria (GNB), such as carbapenem-resistant *Enterobacteriaceae* and *Pseudomonas aeruginosa* (Thomsen et al., 2023; Govindaraj and Vanitha, 2018). It has demonstrated high effectiveness in the treatment of complex abdominal infections, hospital-acquired pneumonia and ventilator-associated pneumonia caused by these pathogens (Tacconelli et al., 2018; Yu et al., 2023).

With the increasing use of ceftazidime/avibactam, its safety profile receives attention. Central nervous system (CNS)-related adverse events are among the most reported severe events in clinical trials and post-marketing reports (Torres et al., 2018; Carmeli et al., 2016). It has been reported that ceftazidime/avibactam is potentially associated with nervous system disorders in patients older than 65 years and with long treatment courses (Guo et al., 2022). Importantly, the occurrence of adverse events in the CNS is often concentration dependent (Martinez-Rodriguez et al., 2001; Chuang et al., 2004). As ceftazidime and avibactam are mainly eliminated by kidney, patients with chronic kidney disease (CKD) are much more susceptible to high-concentration exposure because of decreased renal clearance. Moreover, these patients frequently receive continuous renal replacement therapy (CRRT) when admitted to the ICU (Aloy et al., 2020). There are limited data regarding the pharmacokinetic parameters for CKD patients during CRRT and after its cessation, and it is difficult to dose ceftazidime/avibactam in these patients (Yu et al., 2023; Collignon et al., 2024). Moreover, although CNS adverse events associated with ceftazidime/avibactam have been reported, the threshold concentration is unknown.

Therefore, this case reports the pharmacokinetic parameters of ceftazidime and avibactam in an elderly CKD patient during and after CRRT. Moreover, CNS adverse events caused by ceftazidime/avibactam were observed in this patient, which allowed us to estimate the possible associations between CNS adverse effects and drug exposure.

## 2 Case presentation

A 91-year-old male patient (body weight 60kg, height 168 cm) was admitted to the hospital due to severe COVID-19 infection more than 1 year prior. Owing to disturbances in consciousness and low oxygen saturation, the patient was transferred to the intensive care unit (ICU) for monitoring after admission. The patient had a long stay in the ICU because his respiratory function could not recover, and mechanical ventilation could not be removed. He had a history of hypertension, gout, chronic kidney disease (G3b--G4, classified by Kidney Disease Improving Global Outcomes), nephrolithiasis and kidney cysts for more than 30 years. During hospitalization in the ICU, he experienced left cerebral hemorrhage (6 months after admission), extensive left cerebral infarction (6 months after admission), subfalcine herniation, arrhythmia (paroxysmal atrial fibrillation) and gastrointestinal bleeding. He received CRRT for approximately 5 times every month due to renal failure. In our hospital, the duration of CRRT is usually 72 h as filters need to be replaced after such a period, and the physician would decide whether a next round of CRRT is needed.

After 11 months of hospitalization in the ICU, the patient developed hospital-acquired pneumonia, and the pathogen was carbapenem-resistant *Klebsiella pneumoniae,* which was identified via sputum culture. The patient experienced an increase in body temperature and infection indicators. The initial antibiotic regimen was 50 mg of tigecycline every 12 h combined with 1.00 g/0.25 g of ceftazidime/avibactam every 8 h over 1-h infusion (The first day of ceftazidime/avibactam administration is defined as Day 1). The patient was undergoing CRRT, and the dosing of ceftazidime/avibactam was selected on the basis of the most used recommendation (Nekidy et al., 2023). The patient was subjected to CRRT in continuous veno venous hemofiltration (CVVH) mode with a dose of 38.6 mL/kg/h (a post-dilution replacement fluid rate of 2,000 mL/h, and an ultrafiltration rate of 50 mL/h). Other related medications, body temperature and laboratory tests of the patient are detailed in Table 1. There was a significant decrease in body temperature and inflammatory marker levels over the next several days.

**TABLE 1:** Treatment course and laboratory test results.

DAY	Day-3	Day-2	Day-1	Day1	Day2	Day3	Day4	Day5	Day6	Day7	Day8	Day9	Day10	Day11
Event						TDM			Occasional tremor can be seen				Severe AE and TDM	
CRRT														
CAZ/AVB														
Tigecycline														
Voriconazole														
Methylprednisolone														
Amiodarone														
24-hour urine output (mL)			850	800	1050	1650	1150	800	1300	1350	2150	1700	1000	1400
24-hour fluid input/output (mL)			1807/1710	2055/1430	2804/1800	1924/2721	1340/2794	1775/1410	2627/1760	2534/1910	2430/2660	2379/2350		
hsCRP (mg/L)	23.1	23.3	73.2	182.8	198.2	205.2	136.4	79.5	39.2	28.4	19	12.2	10.4	9.6
PCT (ng/ml)	0.26	0.21	0.37	0.63	0.74	0.82	0.64	0.62	0.51	0.48	0.41	0.31	0.28	0.21
IL-6 (pg/ml)	-	-	-	1111	820.8	173.5	116.3	46.5	34.6	29.1	30.2	21.8	22.2	53.2
WBC (10^9^/L)	14.4	12.9	13.1	13.2	11.3	13.5	10.3	11.3	11.4	12.4	10.3	7.1	6.9	5.6
N%	90.1	92	88.3	90.2	91.6	94.7	92.8	91.4	91.4	93.1	93.4	90.1	90.1	87.6
T	37.8	37.8	38.3	38	39	36.8	37.2	37.3	37.2	37.1	37.1	36.9	36.8	37.9
SCR (umol/L)	95	60	79	118	150	161	64	88	112	123	129	128	119	110

Note: The colored sections in the table represent the use of items on the specified days. CRRT, continuous renal replacement therapy; CAZ/AVB, ceftazidime/avibactam (1.25 g q8h via 1-h infusion, the dose was not changed from Day1 to Day9); TDM, therapeutic drug monitoring; AE, adverse event; hsCRP, hypersensitive C-reactive protein; PCT, procalcitonin; IL-6, interleukin-6; WBC, white blood cell; N%, neutrophil granulocyte; T, body temperature; SCR, serum creatinine.

**FIGURE 1:**
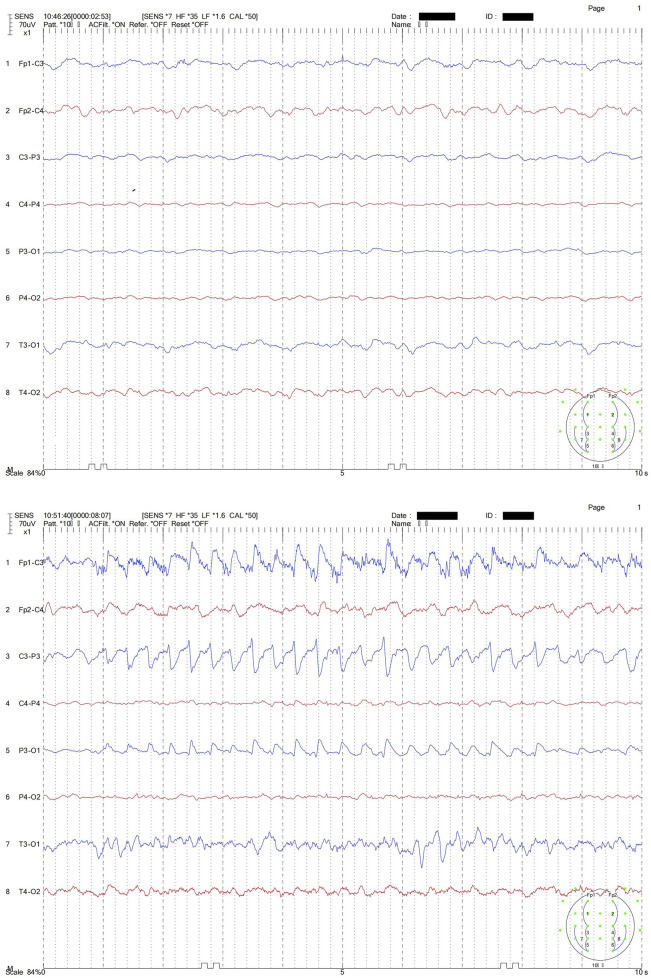
EEG of the patient. Note: The top half shows the abnormal slow waves in EEG; the bottom half shows the synchronized motion artifacts in EEG during the patient’s left upper limb rhythmic involuntary flapping.

The patient underwent TDM with ceftazidime/avibactam using CRRT (CVVH model) on day 3 after the sixth dose. CRRT was ceased on day 4 but the dose of ceftazidime/avibactam was not changed, which may be excessive for CKD patients without CRRT. Occasional tremors and increased heart rates can be observed in this patient over the next several days. On Day 10, the patient experienced severe tremor of the whole body and an increased heart rate above 100 beats per minute. In particular, the patient’s left upper limb presented with involuntary convulsions. The patient’s Glasgow Coma Scale (GCS) score at that time was 4 + T + 5, which was the same as before ceftazidime/avibactam use. The patient’s creatine clearance (CrCL) during the period when patient experienced severe tremor and elevated heart rate was difficult to estimate due to instable renal function, but the high serum creatine level (1.35 mg/dL) indicated low drug clearance. The moderate to highly abnormal EEG on Day 10 revealed that the α waves could not be traced and that moderate low-amplitude fast waves were visible, with low to moderate 4–5 Hz medium-amplitude θ waves and more 2–3 Hz medium to high-amplitude unilateral δ waves and irregular recombination δ diffuse distributions, with basic symmetry on both sides (Figure 1, top half). During the process, the patient’s left upper limb was involuntarily flapped, and EEG revealed synchronized motion artifacts with similar rhythms (Figure 1, bottom half, blue line in the figure). We hypothesized that this was a CNS adverse reaction associated with ceftazidime/avibactam. The Naranjo score was used to evaluate the relationship between the drug and adverse events, and the score was 8 (details in Supplementary Table S1), which indicated a “Probable” relationship. Ceftazidime/avibactam was suspended, and a midazolam injection was administered to alleviate symptoms. We collected the blood samples at 17 h and 24 h after the last dose. The patient still presented mild CNS adverse reactions 24 h after the last dose, which may provide information about possible relationship between concentrations and CNS adverse reactions.

## 3 PK parameter calculation

Blood samples were collected at predetermined time points, and the plasma was separated via centrifugation at 3,000 *g* for 10 min and then stored at −80°C before analysis. The concentrations were determined via a validated liquid chromatography–tandem mass spectrometry (Ab Sciex Triple Quad™ 4500 LC‒MS/MS) method (Cheng et al., 2022). The pharmacokinetics of both ceftazidime and avibactam could be considered one-compartment models with first-order elimination (Li et al., 2024). Therefore, PK parameters for ceftazidime and avibactam during and after CRRT were estimated via the Equations 1–5 below.

$$C=C_{peak}e^{-kt}$$ (1)

$$lgC=-\frac{k}{2.303}t+\mathrm{lg}C_{peak}$$ (2)

$$\mathrm{lg}C_{1}-\mathrm{lg}C_{2}=-\frac{k}{2.303}\left(t_{1}-t_{2}\right)$$ (3)

$$t_{\frac{1}{2}}=\frac{0.693}{k}$$ (4)

$$k_{Total}=k_{Non-CRRT}+k_{CRRT}$$ (5)

where *k* is the elimination rate constant, *t*_*1/2*_ is the half-life, *C*_*peak*_ is the peak blood drug concentration after infusion, *t*_*n*_ represents the corresponding time and *C*_*n*_ represents the corresponding blood drug concentration. We assumed that Non-CRRT drug clearance remained constant during these days.

The sampling times after the 6th (undergoing CRRT) and 27th (after the cessation of CRRT for 6 days) administrations and the corresponding concentrations at the respective time points are listed in Table 2. The PK parameters of ceftazidime and avibactam are also listed in Table 2. CRRT accounted for 84.9% of the total elimination of ceftazidime and 77.1% of the total elimination of avibactam during CRRT treatment days.

**TABLE 2:** Pharmacokinetic parameters of ceftazidime/avibactam during and after CRRT.

	Ceftazidime		Avibactam	
	Day3 (on CRRT)	Day10 (off CRRT)	Day3 (on CRRT)	Day10 (off CRRT)
Time of sampling (after dose) and concentrations (mg/L)	4 h: 63.6 8 h: 36.5	17 h: 127 24 h: 109	4 h: 16.0 8 h: 10.7	17 h: 24.1 24 h: 20.6
Extrapolated C_peak_ (mg/L)	114.7	181.9	24.5	35.8
k_Total_ (h^-1^)	0.139	-	0.100	-
k_Non-CRRT_ (h^-1^)	-	0.0210	-	0.0229
k_CRRT_ (h^-1^)	0.118		0.0771	
T_1/2_ (h)	4.99	33.0	6.89	30.3

Note: k, elimination rate constant; T_1/2_, half-life. On Day 3, the results were under the CRRT; on Days 10, the results were obtained after the cessation of CRRT, for 6 days and 24 h after the cessation of administration.

## 4 Discussion

This case reported the pharmacokinetics of ceftazidime and avibactam during and after CRRT in an elderly patient with CKD and, for the first time, related the plasma concentration of ceftazidime/avibactam to its adverse effects on the CNS. The PK parameters revealed that after the withdrawal of CRRT, the total drug clearance decreased, and the drug accumulated, leading to a severe increase in drug exposure in patients. Clinical and EEG manifestations of CNS adverse events caused by ceftazidime/avibactam accumulation were presented, which occurred when the total plasma concentrations of ceftazidime and avibactam were above 109 and 20.6 mg/L, respectively.

There is limited clinical evidence regarding the use of ceftazidime/avibactam in patients undergoing CRRT. Comprehensive data on its pharmacokinetics under CRRT (CVVH) conditions remain insufficient. An intro study found that for avibactam, CL of CRRT ranged from 15.07 to 18.82 mL/min for CVVH and CVVHD (Alarcia-Lacalle et al., 2023). Wenzler’s study evaluated the pharmacokinetics in a critically ill patient undergoing CVVH (1.25 g q8h for ceftazidime/avibactam; the blood and ultrafiltration flow rates were fixed at 200 mL/min and 2 L/h, respectively, and replacement fluid was added prefiltration for CVVH). Drug concentrations and postfiltration and ultrafiltrate concentrations were measured. The t_1/2_ for ceftazidime was 6.07 h, whereas that for avibactam was 6.78 h. The k value of ceftazidime was inferred to be 0.114 h^−1^, whereas the k value of avibactam was inferred to be 0.102 h^−1^. These results, especially those of avibactam, are similar to ours. This study also revealed that CVVH accounted for 57.1% of the total clearance of ceftazidime and 54.3% of the total clearance of avibactam, as calculated on the basis of postfiltration and ultrafiltrate concentrations (Wenzler et al., 2017). Soukup et al. reported a case of clearance under CVVHDF mode (2.5 g, q8 h), with half-lives of 5.17 h and 5.92 h, respectively. However, the CRRT clearance rate was not calculated separately (Soukup et al., 2019). Zhang et al. reported a clearance case under CVVHD mode (2.5 g q12 h), with half-lives of 4.99 h and 9.93 h, respectively, but CRRT clearance rates were also not mentioned in the report (Zhang et al., 2022). A recent study including 4 CVVHDF patients found that the median total clearance and volume of distribution were 4.54 L/h and 73.2 L for ceftazidime and 10.5 L/h and 102 L for avibactam, respectively (O'Jeanson et al., 2025). Although the variance of parameters from different studies are not large, the CRRT modality has a significant effect on drug clearance, and those cases are less informative for CVVH patients. Moreover, there are many potential factors that would influence drug clearance, such as CRRT setting and patients’ residual renal function. This case has added pharmacokinetic data of ceftazidime and avibactam in CRRT patients, which would benefit future dosing in these patients. Additionally, we used first-order elimination equation and a two-point sampling strategy to estimate the PK parameters during CRRT. The steady status is not required for this method and it would be more practical in clinical scenarios. In this case, the half-lives of ceftazidime and avibactam under CRRT were 4.99 and 6.89 h, respectively. These results were similar to previous report in patients with various model of CRRT. When CRRT was ceased, the half-lives of ceftazidime and avibactam were prolonged significantly. Although the parameters of this case have large uncertainty, it is important to reduce the dose after the withdrawal of CRRT.

Encephalopathy, seizure and myoclonus are the most common CNS adverse events associated with ceftazidime/avibactam, and their occurrence may be related to drug concentrations (Guo et al., 2022). However, the threshold concentration is unknown. Fortunately, some cases of ceftazidime-induced encephalopathy are related to drug exposure. Suda reported a case of nonconvulsive status epilepticus as an adverse reaction to ceftazidime. The ceftazidime dosing regimen was 1000 mg q12 h, and the peak plasma concentration collected after the adverse reaction occurred was 105.2 μg/mL (Vannaprasaht et al., 2006). Chuang et al. reported a case of ceftazidime-induced Creutzfeldt‒Jakob-like EEG, with a peak drug concentration reaching 480 μg/mL before hemodialysis (Chuang et al., 2004). However, Guo et al. noted that the combination of ceftazidime and avibactam is associated with a greater likelihood of encephalopathy and myoclonus than ceftazidime alone, and the threshold would also be different (Guo et al., 2022). The clinical and EEG manifestations of CNS adverse events caused by ceftazidime/avibactam accumulation were presented in our patient. When the ADR occurred, the plasma concentrations of ceftazidime and avibactam were above 109 and 20.6 mg/L, respectively. Subsequent midazolam was administered to control for adverse events, and further ADR-exposure relationships could not be observed. Our study provides potential information for the possible relationship between concentrations of ceftazidime/avibactam and CNS adverse reactions. Continuous infusion or prolonged infusion is a promising dosing strategy for ceftazidime/avibactam, as it may achieve PK/PD target at a lower dose than intermittent infusion (Han et al., 2024). It should be noted that some studies have reported similar drug levels in patients without CNS events (Fresan et al., 2023; Yasmin et al., 2023), and the link to CNS toxicity in this case is speculative.

This case study has several limitations. Although the Naranjo score indicated a possible relationship between ceftazidime/avibactam use and ADR, the polypharmacy and underlying diseases would be confounding factors. The PK parameters were calculated based on limited samples, which may cause random error in parameter estimation. Considering the additional uncertainty in clinical setting, the estimated PK parameters should be interpreted with caution. The eGFR of patients, especially post-CRRT, was difficult to be estimated, which would undermine the strength of clearance distribution. With respect to the exposure‒ADR relationship, the drug concentrations in cerebrospinal fluid may be relatively important. However, we did not perform lumbar puncture to obtain and measure drug concentrations in the cerebrospinal fluid due to safety and benefit-risk evaluations, and this should be investigated in future studies.

## 5 Conclusion

This case reported the pharmacokinetic parameters of ceftazidime/avibactam in an elderly patient during and after CRRT. CRRT constituted approximately 80% of the total clearance of ceftazidime and avibactam. Owing to the removal of CRRT and elevated plasma drug concentrations, excessive drug exposure has caused severe CNS adverse events, as proven by clinical manifestations and EEG. The pharmacokinetic data of this case could benefit future dosing in these patients and finding a safety threshold. More data about the pharmacokinetics in CRRT patients are needed, as well as the upper limit of exposure to ceftazidime/avibactam.

## Data Availability

The original contributions presented in the study are included in the article/Supplementary Material, further inquiries can be directed to the corresponding authors.
